# Dysbiosis and extraintestinal cancers

**DOI:** 10.1186/s13046-025-03313-x

**Published:** 2025-02-07

**Authors:** Ruishan He, Pingqian Qi, Linzhen Shu, Yidan Ding, Peng Zeng, Guosheng Wen, Ying Xiong, Huan Deng

**Affiliations:** 1 The MOE Basic Research and Innovation Center for the Targeted Therapeutics of Solid Tumors, Affiliated Rehabilitation Hospital, Jiangxi Medical College, Nanchang University, No. 133 South Guangchang Road, Nanchang, Jiangxi Province 330003 China; 2 Department of Breast Surgery, Jiangxi Armed Police Corps Hospital, Nanchang, China; 3 Department of General Medicine, The Second Affiliated Hospital, Jiangxi Medical College, Nanchang University, Nanchang, 330031 Jiangxi China; 4 Tumor Immunology Institute, Nanchang University, Nanchang, 330006 Jiangxi China

**Keywords:** Microbiota, Dysbiosis, Extraintestinal cancers, Carcinogenesis, Inflammation

## Abstract

The gut microbiota plays a crucial role in safeguarding host health and driving the progression of intestinal diseases. Despite recent advances in the remarkable correlation between dysbiosis and extraintestinal cancers, the underlying mechanisms are yet to be fully elucidated. Pathogenic microbiota, along with their metabolites, can undermine the integrity of the gut barrier through inflammatory or metabolic pathways, leading to increased permeability and the translocation of pathogens. The dissemination of pathogens through the circulation may contribute to the establishment of an immune-suppressive environment that promotes carcinogenesis in extraintestinal organs either directly or indirectly. The oncogenic cascade always engages in the disruption of hormonal regulation and inflammatory responses, the induction of genomic instability and mutations, and the dysregulation of adult stem cell proliferation. This review aims to comprehensively summarize the existing evidence that points to the potential role of dysbiosis in the malignant transformation of extraintestinal organs such as the liver, breast, lung, and pancreas. Additionally, we delve into the limitations inherent in current methodologies, particularly the challenges associated with differentiating low loads gut-derived microbiome within tumors from potential sample contamination or symbiotic microorganisms. Although still controversial, an understanding of the contribution of translocated intestinal microbiota and their metabolites to the pathological continuum from chronic inflammation to tumors could offer a novel foundation for the development of targeted therapeutics.

## Background

Cancer remains one of the leading causes of morbidity and mortality worldwide [1]. As a complex cascade, the progression of malignant tumors is shaped by a combination of intrinsic and extrinsic factors [2]. The carcinogenic roles of certain viruses, including human papilloma virus (HPV), hepatitis C virus (HCV), and Epstein–Barr virus (EBV), have been firmly established through both animal models and clinical trials. Accumulating evidence further highlights the potential involvement of bacteria and fungi in the process of malignant transformation [3, 4].

The human gastrointestinal tract harbors a vast community of over 100 trillion bacteria [5], which play a crucial role not only in normal development and physiological homeostasis [6, 7] but also in the initiation and progression of various cancers. Notably, *Helicobacter pylori*, the most well-known driver of gastric cancer [8], infects up to 43.1% of the global population [9]. Similarly, *Escherichia coli*, a pathogenic bacterium that establishes a symbiotic relationship with humans from infancy, has been shown to induce DNA damage in intestinal epithelial cells (IECs) [10]. Furthermore, in macrophage-deficient mouse models, *Candida tropicalis* has been observed to recruit myeloid-derived suppressor cells (MDSCs), thereby promoting the development of colorectal cancer (CRC) [11].

Gut dysbiosis, characterized by an imbalance between probiotic and pathogenic microorganisms, has been increasingly associated with the development of gut precancerous lesions, including irritable bowel syndrome (IBS) [12], inflammatory bowel disease (IBD) [13], colorectal polyps [14], and CRC [15]. Recent advances in the application of next-generation sequencing have significantly enhanced our understanding of the composition and functional dynamics of microbial community, shedding light on the intricate connections between the gut microbiome and the pathogenesis of extraintestinal cancers [16–19]. The gut microbiota profiles of cancer patients exhibit marked differences from those of healthy individuals. Moreover, accumulating evidence suggests that many microorganisms detected within tumor tissue may originate from the intestinal microbiota [20–23]. Despite these advances, the precise pathogenic mechanisms remain largely unknown, particularly regarding how the gut microbiota disrupts the intestinal barrier, disseminates through the circulatory system, colonizes distant organs, and ultimately exerts carcinogenic effects on parenchymal cells.

This review highlights recent advancements in understanding the close association between gut dysbiosis and tumorigenesis in extraintestinal organs, including the liver, breast, lung, and pancreas. A particular emphasis is placed on elucidating the molecular mechanisms by which the gut microbiota contributes to the pathological continuum from chronic inflammation and dysplasia to primary and metastatic tumors. Given the substantial evidence indicating that a significant proportion of intratumoral microbiota may represent translocated gut microbiota, we also explore their potential contributions to the establishment of the tumor microenvironment (TME). Additionally, we critically evaluate the advantages, limitations, and future directions of diagnostic technologies for detecting and characterizing extraintestinal tumors.

### Relationship between the intestinal microbiota and extraintestinal cancers

#### Intestinal barrier impairment

Clinical evidence underscores that dysbiosis is a serious threat to the integrity of the gut barrier, a phenomenon temporally linked to the pathogenesis of various extraintestinal cancers [24]. The human body employs a sophisticated system to maintain gut homeostasis, which is essential for resisting the invasion of pathogenic microbiota and their detrimental metabolites during host-microbe interactions [25]. The gut barrier is a multifaceted structure comprising two primary components: (1) a physical barrier, which includes the mucus layer, IECs/intercellular junctions and endothelial cells of blood vessels; and (2) a functional barrier, primarily consisting of antibacterial proteins and intestinal alkaline phosphatase [25–27]. The physical barrier, also known as the mechanical barrier, plays a pivotal role in preventing the translocation of luminal microbiota and metabolites across the intestinal epithelium under homeostatic conditions [28, 29].

The outer mucus layer, predominantly formed by mucins, acts as the first line of defense, shielding epithelial cells from pathogens while fostering a symbiotic relationship with commensal microbiota [29]. Beneath this mucus layer lies a selectively permeable barrier consisting of a diverse array of epithelial cells, including enterocytes, enteroendocrine cells, Paneth cells, tuft cells, goblet cells, microfold cells, and intestinal stem cells [30, 31]. These epithelial cells are interconnected by specialized junctional structures such as tight junctions (TJs), adherens junctions, desmosomes, and gap junctions. TJs, located at the apical region of epithelial cells, constitute the majority of intercellular junctions and are predominantly composed of structural proteins like occludin, claudins, junctional adhesion molecules (JAMs), and tricellulin [32]. Additionally, functional proteins such as zonula occludens-1 (ZO-1), ZO-2, and ZO-3 regulate paracellular permeability, ensuring the selective passage of molecules and microorganisms to maintain intestinal barrier integrity [33–35].

The intestinal vasculature is lined by the gut vascular barrier (GVB), a specialized network of endothelial cells interconnected by TJs. This barrier is further supported by pericytes and enteric glial cells, ensuring the selective exchange of substances between the intestinal lumen and bloodstream [28, 36]. The integrity of GVB is maintained by specific proteins, including plasmalemma vesicle-associated protein-1 (PV-1), which plays a critical role in modulating barrier permeability and has been implicated in the progression of various diseases [37].

Bjarnason et al*.* first coined the term “leaky gut” to describe a pathological state of the intestine characterized by increased permeability, initially observed in individuals with excessive alcohol consumption [38]. This condition may enable the translocation of microbiota and/or harmful metabolites into the bloodstream, potentially triggering chronic immune responses and predisposing inflamed organs to carcinogenesis. Intestinal barrier damage can be categorized into endogenous and exogenous subtypes [39]. Lipopolysaccharide (LPS), also known as endotoxin, is primarily derived from the outer membrane of gram-negative bacteria in the gut and exhibits extensive biological activities. Even at low concentrations, LPS can elicit a potent inflammatory response by binding to Toll-like receptor 4 (TLR4) and its coreceptor myeloid differentiation protein 2 on immune cells. This interaction activates the transcription factor NF-κB, leading to the production of proinflammatory cytokines such as tumor necrosis factor-α (TNF-α), interleukin-6 (IL-6), and IL-1β [40, 41]. LPS disrupts intestinal barrier function by activating the TLR4/NF-κB signaling pathway, causing significant mislocalization of the ZO-1 protein [42, 43].

Furthermore, inflammatory cytokines like interferon-γ (IFN-γ) and TNF-α can exacerbate intestinal permeability through synergistic mechanisms, including the induction of IECs death and the internalization of transmembrane proteins [39]. IFN-γ also activates the phosphoinositide 3-kinase/protein kinase B (PI3K/Akt) pathway, prolonging the NF-κB response, which reduces occludin expression and increase TJ permeability [44]. Meanwhile, TNF-α exerts its effects by activating myosin light chain kinase (MLCK) [45], resulting in cytoskeleton rearrangement, contraction of the perijunctional actomyosin ring, and ultrastructural alterations in TJs [46, 47].

Several external factors, including transient exposure to pathogenic microbes, alcohol consumption, drugs, and unhealthy dietary habits, can adversely affect intestinal permeability [48]. Pathogenic bacteria often utilize the type III secretion system (T3SS) to inject effector proteins into eukaryotic cells, disrupting the host cytoskeleton [49]. *Salmonella*, a common foodborne pathogen, is typically transmitted through contaminated food or water [50]. Mutations in the *Salmonella* SPI2 T3SS result in the formation of enlarged *Salmonella*-containing vacuoles within IECs. SPI2 T3SS effectors interact with the host’s microtubule network and intracellular transport systems, facilitating the apical-to-basolateral migration of these vacuoles. This process may contribute to increased intestinal permeability [51]. However, the precise mechanisms by which SPI2 T3SS effectors modulate the gut barrier, particularly their impact on epithelial TJ proteins, remain poorly understood and warrant further investigation.

Probiotic supplementation has demonstrated beneficial effects on intestinal barrier integrity across diverse age groups [52]. For example, feeding preterm infants formula supplemented with *Bifidobacterium lactis* (2 × 10⁷ CFU/g dry milk) for 30 days significantly decreases intestinal permeability [53]. Similarly, in middle-aged IBS patients (approximately 48 years old), the administration of *Bifidobacterium longum BB536* and *Lactobacillus rhamnosus HN001*, combined with vitamin B6, has been shown to enhance gut microbiota diversity, decrease intestinal permeability, and alleviate clinical symptoms. Gut microbiota analysis revealed that the abundance of probiotic *Lactobacillus*/*Bifidobacteria* populations correlates positively with increased levels of short-chain fatty acids (SCFAs), both of which favor the maintenance of gut barrier integrity [54]. In elderly populations, a clinical trial demonstrated that supplementation with a probiotic mixture (2.0 × 10^10^ CFU of *Lactobacillus paracasei* HII01; 2.0 × 10^10^ CFU of *Bifidobacterium* *breve*; 1.0 × 10^10^ CFU of *Bifidobacterium longum*) promotes gut homeostasis [55]. These findings underscore the potential of probiotic-based interventions in repairing and preserving intestinal barrier function across different life stages.

Disruption of TJs is a key factor in alcohol-associated organ injuries [56]. Alcohol-induced dysbiosis activates monocytes and macrophages in the intestinal lamina propria to produce TNF-α, which binds to TNF-receptor I on intestinal epithelium and disrupts TJs through the activation of MLCK [57]. Antibiotic use, which affects individuals across all life stages, significantly disrupts the delicate balance of the gut microbiota and impairs host physiological functions [58, 59]. This disruption is akin to the “eradication” of microbial species, resulting in reduced microbial diversity and altered abundance within specific taxa, thereby indirectly compromising intestinal barrier function. Different antibiotics target specific microbial populations, impairing various components of the barrier. Antibiotic-induced disturbances reduce goblet cell function and mucus layer thickness in animal models. Alterations in pathogen-associated molecular pattern (PAMP) concentrations affect the secretory functions of IECs [60].

Recent studies have also explored the impacts of dietary fat on gut microbial composition, revealing that high-fat diets reduce the abundance of beneficial microbes that maintain intestinal barrier integrity while promoting the growth of potentially detrimental microbes [61, 62]. These alterations may negatively impact barrier function through metabolic pathways, such as the upregulation of lysophosphatidylcholine (LPC) and lysophosphatidic acid (LPA) [63]. In mice with intestinal epithelial cell-specific deficiency of fucosyltransferase 2 (Fut2), gut microbiota diversity is significantly reduced, contributing to increased LPC production. Both in vivo and in vitro experiments have demonstrated that LPC not only significantly reduces the number of mucus-secreting goblet cells but also inhibits the expression of TJ proteins such as ZO-1 and occludin [64].

**Fig. 1:**
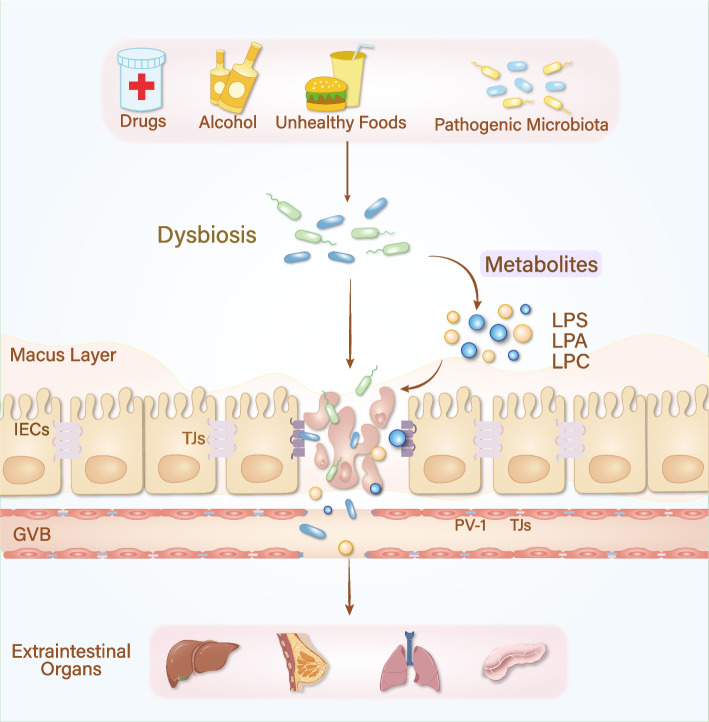
Life patterns, dysbiosis, and extraintestinal cancer. Exogenous detrimental factors, including pharmaceutical agents, alcohol consumption, high-fat dietary habits, and external pathogenic microbial exposure, contribute to intestinal barrier dysfunction and initiate a pathological continuum from chronic inflammation to metastatic cancer in distant organs such as the liver, breast, lung, and pancreas

Given the profound influence of diet on the host microbiota, researchers have analyzed the diversity of food-associated microbiomes and integrated these findings with 19,833 human metagenomes [65]. It revealed significant overlap at the species and strain levels, highlighting the potential for therapeutic applications of food-derived microbiomes in clinical practice. Dysbiosis is influenced by a wide range of internal and external factors, resulting in diverse and complex mechanisms that regulate intestinal barrier permeability. This, in turn, promotes the spread of chronic inflammation, a well-established risk factor for tumorigenesis, making intestinal barrier dysfunction a potential predictor of cancer progression (Fig. 1).

Clinical data indicate that up to 80% of patients with cirrhosis eventually develop hepatocellular carcinoma (HCC). This oncogenic progression, also termed the “leaky gut” cascade, is often accompanied by gut dysbiosis and increased intestinal permeability [16]. The compromised intestinal barrier allows pathogens and their metabolites to translocate from the intestinal lumen into the portal system, where they land on the liver and initiate a pathological continuum from hepatitis and fibrosis to cirrhosis and, ultimately, HCC [66]. Disruption of GVB represents a critical rate-limiting step in this cascade. Spadoni et al*.* demonstrated that excessive colonization by intestinal bacterial pathogens, such as *S. typhi,* induces GVB damage, characterized by upregulated expression of PV-1, thereby promoting the systemic spread of bacteria and their toxic metabolites [67, 68]. In support of this hypothesis, Bertocchi et al*.* reported that *E. coli* (C17 strain) employs a virulence factor-dependent T3SS system to attack GVB in CRC patients, facilitating its entry into the bloodstream and subsequent extravasation into the liver. These translocated bacteria recruit immune cells to establish a premetastatic niche, fostering the development of metastases [69].

Metabolic-associated fatty liver disease (MAFLD) is a leading cause of HCC [70]. Increased intestinal barrier permeability, driven by intestinal inflammation, significantly exacerbates liver damage. Cheng et al*.* provided compelling evidence that mice fed a high-fat diet combined with dextran sulfate sodium (DSS) treatment exhibited more severe hepatic steatosis, inflammation, and fibrosis compared to controls fed a high-fat diet alone [71]. These findings underscore the role of DSS-induced colitis in aggravating hepatic degeneration. Further investigation revealed downregulated expression of epithelial ZO-1 and claudin-1 in DSS-treated mice, which facilitated bacterial invasion. Increased macromolecular permeability and elevated expression of endothelial PV-1 indicated GVB damage, promoting bacterial translocation and accelerating the progression of nonalcohol-associated steatohepatitis in the liver [71]. These results generally suggest that intestinal barrier injury acts as a critical mediator of liver damage and serves as an important risk factor for HCC development.

Intestinal barrier dysfunction also plays a significant role in the development of extraintestinal malignancies by modulating immune responses. When the intestinal barrier is compromised, gut-derived PAMPs and bacterial metabolites can enter the liver and subsequently activate TLRs, initiating protumorigenic immune responses [72]. Transplanting fecal microbiota from HCC patients into mice demonstrated that *Klebsiella pneumoniae* enhances the activity of macrophage-derived gelatinase in the colon, resulting in intestinal barrier dysfunction. This disruption facilitates the migration of *K. pneumoniae* to the liver, where its surface protein PBP1B binds to TLR4 on HCC cells. This interaction activates the TLR4 signaling pathway, ultimately driving the initiation and progression of HCC [73]. In chronically injured livers, TLR4 acts as a critical driver of HCC progression. Studies have shown that mice with genetically inactivated TLR4 exhibit a significantly lower incidence of HCC in the context of chronic liver injury [74]. TLR4 activation accelerates hepatic stellate cell (HSC) fibrosis via two distinct mechanisms. First, it enhances chemokine secretion (e.g., Ccl2 and Ccl5) by Kupffer cells, and second, it increases HSC sensitivity to transforming growth factor (TGF)-β, thereby accelerating early-stage HCC progression [75]. In advanced HCC, TLR4 expressed on hepatocytes provides survival signals to nontumorous liver cells, maintaining the viability of tumor precursor cells and facilitating further HCC development [74].

In addition, overexpression of TLR4 indirectly modulates the suppressive function of regulatory T cells (Tregs) and recruits Tregs through cytokine signaling [76], creating an immune exclusion barrier within the TME. This barrier prevents the infiltration of proinflammatory immune cells, thereby suppressing antitumor immune responses [76, 77]. Dysregulated activation of the TLR4/MyD88/NF-κB pathway has been identified as a significant risk factor for dysbiosis-associated extraintestinal tumorigenesis [78]. The leaky gut allows the accumulation of microbiota-derived LPS in the liver through the portal circulation. Upon binding to TLR4, LPS activates the MyD88/NF-κB signaling axis, leading to the upregulation of downstream proinflammatory cytokines such as IL-1β, IL-6, and TNF-α [79, 80]. Additionally, LPS directly enhances the migratory capacity of HCC cell lines by increasing the gene expression of IL-8 and TGF-β1 [81].

Dysbiosis also exerts detrimental effects on breast tissues, with accumulating evidence suggesting that infections induced by intestinal pathogenic microbiota can rapidly accelerate breast tumorigenesis [21], likely mediated by intestinal barrier disruption. The intestinal epithelial vitamin D receptor (VDR) is essential for maintaining gut and microbial homeostasis [82]. In conditionally deficient mice (VDR^ΔIEC^), downregulation of ZO-1 indicates impaired TJ function and increased intestinal permeability, followed by significant alterations in gut bacterial abundance and metabolic activity [83]. Notably, compared with their WT littermates, VDR^ΔIEC^ mice develop more breast tumors and harbor gut-specific bacteria, including *Streptococcus pyogenes*, *Streptococcus*, *Lactobacillus*, *Methylobacterium*, and *Atopobium*, within their breast tissue. These findings suggest that intestinal bacteria infiltrate breast tissue through the compromised gut barrier, altering the local microenvironment and elevating cancer risk [83]. Treatment with the probiotic *Lactobacillus plantarum* improved breast VDR expression and restored colonic ZO-1 levels in VDR^ΔIEC^ mice, significantly reducing tumor incidence and size. This highlights the critical role of gut barrier dysfunction in breast cancer (BC) progression and underscores the potential of probiotics as a therapeutic strategy to mitigate cancer risk.

While the precise mechanisms by which gut microbiota translocate to the breast remain poorly understood, the immune system is likely a key mediator in this process. Pathogens may utilize macrophages as vectors to infiltrate deeper tissues, access blood vessels and lymph nodes, and subsequently disseminate to distant organs [84]. Under physiological conditions, the gut‒breast axis facilitates the transfer of maternal microbiota across the gut barrier to the breast, potentially via lymphatic drainage or through dendritic cells (DCs) and macrophages that transport microbial communities from mucosal tissues to lactating mammary glands [85, 86]. These observations suggest that disruptions in gut permeability and microbiota translocation can profoundly impact breast homeostasis. Understanding the mechanisms underlying microbiota dissemination is essential for developing strategies to protect breast health and prevent dysbiosis-related pathologies.

#### Active roles of the intestinal epithelium

Current research on dysbiosis has predominantly focused on the role of pathogenic bacteria in disrupting IECs and promoting malignant transformation. However, accumulating evidence indicates that IECs not only regulate the gut microbiota balance but also influence the homeostasis of distant organs. For instance, mutations in the *Crumbs homolog 1* (*Crb1*) gene in mice lead to increased permeability in both the gut and retina [87], enabling the translocation of gut microbiota such as *Anaerostipes hadrus* and *Bifidobacterium pseudocatenulatum* from the lower gastrointestinal tract to the eye, resulting in secondary retinal degeneration [87]. Similarly, overexpression of dopamine receptors on the intestinal epithelium reduces the abundance of lysozyme-sensitive microbiota, particularly *Lactobacillus*, leading to decreased levels of N-acetyl-lysine metabolites and triggering multiple sclerosis [88]. Additionally, loss of Claudin-7 reduces the diversity of the gut microbiota and increases susceptibility to colitis [89]. Tuft cells, which recognize microbial metabolites like succinate, release IL-25 to modulate antimicrobial peptide expression through the ILC2/IL-13 pathway, thereby shaping the gut microbial landscape [90]. These findings further explain the intricate bidirectional relationship between IECs and the gut microbiota.

Pioneering work by Chandra et al*.* further demonstrated that the deletion of IL-17RA in IECs induces dysbiosis, elevates systemic IL-17 levels, and promotes pancreatic tumor growth [91]. Collectively, these studies reveal the active regulatory role of IECs not only in maintaining gut microbiota balance but also in the progression of dysbiosis-related diseases. This accumulating evidence opens new avenues for research and therapeutic strategies aimed at targeting IECs-microbiota interactions to prevent or treat systemic diseases.

#### Intestinal dysbiosis and extraintestinal cancers

##### Hepatocellular carcinoma

The intricate communication between the liver and gut underpins the impressive capacity of the liver for regeneration. The portal system serves as a critical conduit for metabolic, immune, and neuroendocrine interactions between the gut and liver [92]. It transports intestinal metabolites to the liver, which delivers bile and antibodies back to the gut via the bile duct, establishing a bidirectional relationship [93, 94]. Accumulating evidence highlights the gut‒liver axis as a central mediator linking dysbiosis to liver diseases, including inflammation and HCC [95, 96]. In this context, PAMPs present in the portal vein are key drivers of liver inflammation, as their levels correlate positively with the extent of intestinal barrier damage [93, 97].

Dysbiosis is evident in patients at various stages of chronic liver disease (CLD) and plays a role in tissue damage, fibrosis, hepatic regeneration, and immune responses, all of which contribute to the progression of CLD and subsequent HCC [16]. In a transgenic mouse model, the absence of NLRP6, an inflammasome sensor molecule critical for host-microbial crosstalk at the gut mucosal surface, induces gut dysbiosis, which, in turn, significantly impairs antitumor immune surveillance and accelerates liver carcinogenesis. Bacterial abundance analyses revealed the absence of *Akkermansia muciniphila* in these mice [20]. *A. muciniphila*, a mucin-degrading bacterium, cooperates with endogenous proteases to maintain the physiological thickness of the mucus layer [98]. This finding suggests that dysbiosis caused by NLRP6 deficiency disrupts intestinal TJ barrier, enabling pathogens or toxins to enter the bloodstream and subsequently reach the liver. Moreover, the absence of *Akkermansia muciniphila* is associated with an increased abundance of liver monocytic myeloid-derived suppressor cells (M-MDSCs), likely through a TLR4-dependent mechanism. This promotes M-MDSCs expansion and suppresses T cell populations, further exacerbating tumor progression [20]. These insights underscore the critical role of the gut-liver axis and microbial balance in liver health and disease.

In mice, infection with *Helicobacter hepaticus* (*H.h.*) attacks the gut barrier, facilitating bacterial translocation to the liver. This process triggers the activation and cytoplasmic translocation of high-mobility group Box 1 (HMGB1), a highly conserved DNA-shepherding protein that amplifies inflammation through TLR activation. HMGB1 regulates downstream MAPK and STAT3 pathways, leading to the release of proinflammatory cytokines such as IL-6, TNF-α and TGF-β, which drive malignant transformation from hepatitis to HCC [99]. HMGB1 plays a central role in liver inflammation, fibrosis, and tumorigenesis by exacerbating inflammatory responses during stress [100].

In cirrhosis patients, translocated gut bacteria, including *Stenotrophomonas, Roseburia, Sphingobiomonas,* and *Psychrobacter*, have been detected in the liver. These bacteria induce significant transcriptional changes, such as the activation of fibrotic and inflammatory pathways and the upregulation of T cell exhaustion markers, thereby mediating cancer-related immunosuppressive circuits [20]. Compared with healthy individuals, HCC patients exhibit a marked reduction in gut microbial composition and abundance. These changes are characterized by a decline in SCFA-producing bacteria and an increase in LPS-secreting genera, creating a proinflammatory environment that promotes disease progression [101, 102].

Microbiota-targeted interventions have shown promise in attenuating liver-related diseases. For example, urolithin A has been demonstrated to enrich certain probiotics, including *Bacteroides sartorii*, *Parabacteroides distasonis*, and *Akkermansia muciniphila*. This phenomenon occurs through the upregulation of urinary protein 1, which suppresses endoplasmic reticulum stress and alleviates alcohol-induced metabolic disorders. These findings position urolithin A as a potential therapeutic and preventive candidate for alcoholic liver disease (ALD) [103]. Such strategies highlight the potential of targeting the gut-liver axis to treat and prevent liver diseases.

In addition to the direct role of dysbiosis in driving the pathological cascade of liver damage, bacterial metabolites are integral to this chain reaction. In mouse models, fed a high-cholesterol diet, dysbiosis is linked to elevated levels of taurocholic acid (TCA) and decreased 3-indolylpropionic acid (3-IPA), both of which correlate with the severity of liver fibrosis. This metabolic imbalance fosters the abnormal accumulation of lipids and inflammatory factors such as IL-6 in the liver, thereby promoting fibrosis and, ultimately, carcinogenesis [104].

Obesity-related changes in gut bacteria can increase the production of deoxycholic acid (DCA), which induces a senescence-associated secretory phenotype (SASP) in HSCs, accelerating malignant transformation [105]. Lipoteichoic acid (LTA), a component of the cell wall in gram-positive bacteria, synergizes with DCA to upregulate SASP factors and TLR2 expression in HSCs. Furthermore, LTA stimulates the production of prostaglandin E2 (PGE2) through the COX2 pathway, which suppresses the secretion of antitumor cytokines by hepatic immune cells [106]. These mechanisms highlight the complex interplay between bacterial metabolites and liver pathology, underscoring their role in promoting inflammation, fibrosis, and cancer progression.

In patients with MAFLD-related HCC, Behary et al*.* identified elevated levels of SCFAs, particularly butyrate, in both fecal and serum samples. These SCFAs were found to promote the expansion of circulating Tregs while simultaneously suppressing cytotoxic CD8^+^ T cells. This immune dysregulation fosters immune tolerance and establishes an immunosuppressive microenvironment, thereby driving tumor progression [107].

**Fig. 2:**
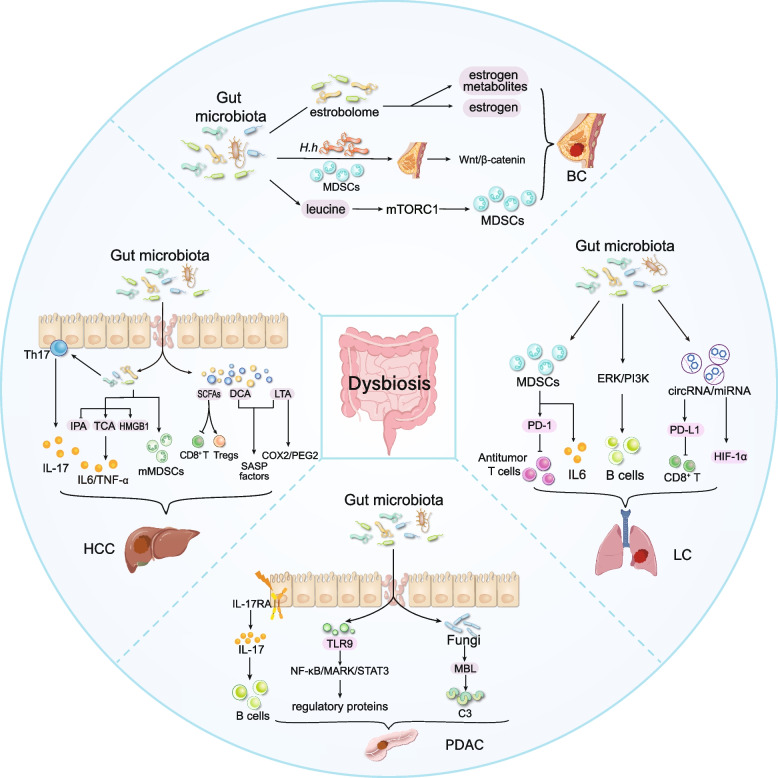
Impact of dysbiosis-related leaky gut on cancer development. Specific pathogen-induced microbial imbalance compromises intestinal barrier integrity, facilitating systemic dissemination of microbiota and their metabolites. They travel through the circulation to land on distant organs and accelerate tumorigenesis through interactions with other microenvironment elements

Given the importance of T cells in the pathogenesis of immune-related diseases, increased frequencies of interleukin-17A (IL-17A)-producing T helper 17 (Th17) cells have been observed in the liver and blood of patients with acute or chronic injury [108]. IL-17A signaling triggers proinflammatory, fibrogenic, and tumorigenic responses in myeloid cells, which, in turn, induce metabolic alterations in hepatocytes affected by nonalcoholic steatohepatitis (NASH) and ALD. These changes accelerate hepatic carcinogenesis [109]. Intriguingly, under normal physiological conditions, Th17 cells reside predominantly in the intestinal lamina propria rather than the liver [110]. This observation suggests that Th17 cells may migrate from the intestine to the liver, where they contribute to the development and progression of CLD (Fig. 2).

The liver plays a pivotal regulatory role in maintaining intestinal homeostasis. One key mechanism involves the secretion of pigment epithelium-derived factor (PEDF), a liver-derived soluble Wnt inhibitor, which prevents excessive proliferation of intestinal stem cells through inhibition of the Wnt/β-catenin signaling pathway, thereby preserving gut epithelial integrity [111]. These findings reinforce the sophisticated, bidirectional communication network between the intestine and liver. Such intricate interactions emphasize the multifaceted nature of the gut‒liver axis, which integrates metabolic and immune regulatory pathways to maintain systemic homeostasis and significantly influences the pathogenesis and progression of both hepatic and intestinal disorders.

##### Breast cancer

The breast microbiome exhibits greater heterogeneity than previously recognized, with emerging evidence highlighting novel mechanisms linking microbial dysbiosis to tumorigenesis, including intestinal microbiota-mediated effects and immune cell recruitment [17]. Intestinal dysbiosis has been proposed as an important risk factor for BC. Parida and colleagues demonstrated that gut colonization by enterotoxigenic *Bacteroides fragilis*, which produces *B. fragilis* toxin, lprogressively increases BC incidence in murine models [112]. In East Asian populations, *Porphyromonadaceae* and *Ruminococcaceae* exhibit an inverse association with BC risk, whereas *Eubacterium ruminantium* functions as a tumor promoter. These taxa, particularly *Porphyromonadaceae* and *Ruminococcaceae*, are closely linked to anti-inflammatory regulation and gut homeostasis [113], indicating the role of chronic intestinal inflammation in disease progression.

Despite growing interest in this field, the molecular mechanisms underlying dysbiosis-related BC remain poorly elucidated. Chronic unresolved inflammation has been implicated in predisposing adult stem cells to malignant transformation [114]. Microbial translocation from the gut to the breast contributes to elevated systemic and local inflammatory responses [21, 22]. Gut infection with the pathogenic bacterium *H.h.* triggers TNF-α-mediated innate immune responses, which can promote tumorigenesis in both the gut and distant breast tissues [115]. Further mechanistic insights were provided by Deng et al*.*, who further demonstrated that MDSCs obtained from an *H.h.*-induced dysbiosis model secrete high levels of Wnt ligands, activating the abnormal translocation of β-catenin from the membrane to the nucleus, resulting in uncontrolled proliferation of breast ductal cells and subsequent carcinogenesis [116].

Probiotic therapy has emerged as a promising frontier in BC management, with growing evidence supporting its therapeutic potential. Clinical studies have demonstrated that probiotic supplementation in patients undergoing chemotherapy can effectively mitigate gut dysbiosis and alleviate chemotherapy-induced cognitive impairment [117]. A novel therapeutic approach combining a synbiotic formulation (comprising eight well-characterized probiotic strains: *Lactobacillus casei*, *L. acidophilus, L. rhamnosus*, *L. salivarius*, *L. reuteri*, *Bifidobacterium lactis*, *B. longum*, and *B. bifidum*) with vitamin D has shown significant enhancement of antitumor immunity in BC patients [118]. These clinical trials support the potential of probiotics as valuable adjuvant agents in comprehensive BC treatment strategies.

Dysregulated estrogen has been proposed as another risk factor for BC. The gut microbiota play a critical regulatory role in regulating systemic estrogen levels [119]. A specialized subset of gut bacteria, collectively termed the "estrobolome", encodes β-glucuronidase and related enzymes, which can facilitate the deconjugation of estrogen metabolites, leading to increased circulating estrogen levels and enhanced estrogen reabsorption, which are well-established clinical markers associated with BC development [120–122]. Interestingly, postmenopausal BC patients exhibit reduced α diversity of IgA-coated gut bacteria compared with healthy individuals. However, this microbial alteration appears to be independent of PGE levels, suggesting that it does not directly influence androgen-to-estrogen conversion pathways. These findings further indicate that the relationship between IgA-coated gut bacteria and BC risk is likely mediated through modulation of the estrogen enterohepatic circulation [123].

In addition to the well-characterized implications of gut dysbiosis, the links between dietary habits, the gut microbiota, and BC progression have been investigated. Notably, consumption of a high-fat diet has been demonstrated to significantly alter gut microbial ecology, particularly through the enrichment of *Desulfovibrio* and MDSCs in both murine models and human subjects. This microbial shift contributes to immunosuppression and promotes tumor progression [124]. This process is mediated by elevated levels of the branched-chain amino acid leucine, which triggers the infiltration and expansion of MDSCs through activation of the mechanistic target of rapamycin complex 1 (mTORC1) signaling pathway [125] (Fig. 2).

In conclusion, substantial evidence supports the concept that pathogenic bacteria and their metabolites can translocate into the circulation, and ultimately land on the breast to influence systemic and local immune responses, resulting in the establishment of a suppressive BC initiation microenvironment [121].

##### Lung cancer

Lung cancer (LC) is the leading cause of cancer-associated death worldwide [1]. The frequent comorbidity of inflammatory bowel disease (IBD) in patients with pulmonary diseases further indicates the existence and clinical relevance of the gut-lung axis (GLA) [126]. The composition of the intestinal bacteria in LC patients is more intricate than that in healthy individuals, with a greater diversity of distinct and specialized pathogenic bacteria, such as *Enterobacteriaceae, Streptococcus,* and *Prevotella*, and alongside reduced populations of probiotic microbes such as *Blautia, Bacteroides, Bifidobacterium,* and *Lachnospiraceae* [18]. Emerging evidence highlights the functional significance of specific microbial species in LC pathogenesis: (1) *Actinobacteria* spp. demonstrate direct antitumor activity through the production of cytotoxic secondary metabolites [127, 128]; (2) *Bifidobacterium* exhibits dual anticancer properties by inhibiting spoilage bacteria growth and eliminating carcinogens, while simultaneously attenuating TNF-α and LPS-induced inflammation [128]; and (3) *Enterococcus* spp. derived pathogens can promote DNA mismatch, abnormal division and proliferation of intestinal parasitic cells through macrophage-mediated pathways [128].

Recent studies have demonstrated that this microbial community affects LC through modulating immune responses and metabolism [129]. The bidirectional regulation of GLA is primarily mediated through dynamic interactions between microbial activity and host immune responses [130]. This crosstalk involves the gut microbiota and pattern recognition receptors (PRRs) of the innate immune system, particularly TLRs, which activate intestinal immune system and trigger the secretion of various cytokines and chemokines. A key mediator in this process is CCL20, a chemokine whose expression is strongly induced by proinflammatory signals and TLR agonists in both pulmonary and intestinal epithelial cells. As a ligand for CCR6, CCL20 orchestrates tissue-specific lymphocyte homing, a critical process for maintaining effective immune surveillance within the GLA [131]. Gut-derived SCFAs have emerged as crucial immunomodulatory metabolites that can enter the bloodstream and reach the bone marrow [132], where they stimulate the proliferation and differentiation of macrophages and DC progenitors. The daughters migrate to the lungs, shaping an immunosuppressive environment that helps regulate allergic airway inflammation and enhances resistance to respiratory infections [133]. These findings demonstrate the profound immunoregulatory capacity of SCFAs in maintaining homeostasis across the gut-lung axis.

Researchers have conducted a Mendelian randomization study to investigate interactions among the human gut microbiota, immune cells, and the development of LC. The findings reveal that immune cells serve as an important bridge between the gut microbiota and malignant lung tumors, particularly small-cell lung cancer (SCLC) [134]. A reduced abundance of *Lactobacillaceae* has been linked to an increase in CD45-positive granulocytic MDSCs, which impair antitumor T cell responses and diminish tumor sensitivity to PD-1 blockade in murine models [135]. Immune checkpoint blockade (ICB) therapy, which disrupts the suppression of antitumor T cells by tumor cells [136], has shown enhanced the efficacy of ICB when combined with the administration of *Clostridium butyricum*, a probiotic that promotes the growth of another probiotic *Bifidobacterium* [137].

The immune cell phenotype “CD20 on IgD^+^CD38^−^ unswitched memory B cells” has been identified as a mediator that regulates the role of *Streptococcus* in SCLC [134]. *Streptococcus* facilitates the upregulation of the extracellular signal-regulated kinase (ERK) and PI3K signaling pathways, which is an early event driving the proliferation, survival, and infiltration of LC cells [138]. These substantial data suggest that an increased abundance of *Streptococcus* may modulate memory B-cell expansion via the PI3K pathway, thereby shaping the immune microenvironment in SCLC [134]. Overall, alterations in the gut microbiota influence immune cell expression, thereby impacting the tumor immune microenvironment, therapeutic outcomes, and prognosis. Consequently, further molecular studies are warranted to elucidate the underlying mechanisms by which microbes synergize with immune cells to influence LC initiation, development, and drug resistance.

Recent study on lung adenocarcinoma (LUAD) has also demonstrated the role of gut microbiome changes in tumorigenesis, primarily through modulation of immune responses. The deletion of the antimicrobial protein *lipocalin-2 (Lcn2)* in mice has been shown to significantly reduce gut microbial diversity, accompanied by an increased relative abundance of *Alistipes* spp. [139], which are known to promote intestinal inflammation and subsequent CRC formation [140]. This localized intestinal inflammation is propagated systemically in an IL-6-dependent manner. IL-6, transcriptionally regulated by NF-κB, activates the STAT3 signaling pathway [141], which is implicated in LUAD pathogenesis. In conclusion, gut microbiota alterations lead to an immunosuppressive TME characterized by an increase in regulatory CD4^+^ T cells and PMN-MDSCs, promoting LUAD via an IL-6-mediated pathway [139].

Noncoding RNAs (ncRNAs) are endogenous RNAs that do not encode proteins but play crucial regulatory roles in various biological processes. Recent investigations have revealed that microRNAs (miRNAs), a prominent subclass of ncRNAs, can modulate bacterial gene transcripts, thereby influencing gut immunity and microbiota-mediated cancer metastasis [142]. Experimental evidence from murine models indicates that gut microbiota depletion alters the circular RNA (circRNA)/miRNA network [143]. The combination of mmu_circ_0000730 and mmu-miR-466i-3p has been shown to counteract the pro-oncogenic effects of SOX9, potentially exerting tumor-suppressive effects through exosome-mediated gene transfer mechanisms [143]. In the setting of non-small cell lung cancer (NSCLC), circ-CPA4 and circPIP5K1A have been identified as key regulators of cancer cell behavior and immune evasion [144, 145]. Circ-CPA4 sequesters let-7 miRNA to enhance PD-L1 secretion by malignant cells. PD-L1 activates both extracellular and intracellular pathways that promote CD8^+^ T cells inactivation, thereby enabling malignant cells to evade immune surveillance [144]. Furthermore, circPIP5K1A contributes to NSCLC progression by sequestering miR-600 and suppressing HIF-1α expression, facilitating tumor growth and metastatic potential [145]. These findings highlight the involvement of gut microbiota in posttranscriptional gene regulation through ncRNA networks, establishing a complex interplay between intestinal homeostasis and NSCLC pathogenesis (Fig. 2).

##### Pancreatic ductal adenocarcinoma

Pancreatic ductal adenocarcinoma (PDAC), with accounts for over 90% of pancreatic malignancies, continues to exhibit dismal clinical outcomes with a 5-year survival rate below 10% [146]. Unfortunately, current therapeutic paradigms, including both adjuvant and neoadjuvant approaches, demonstrate limited efficacy while carrying substantial toxicity burdens. A critical knowledge gap persists in the precise elucidation of modifiable risk factors, significantly hindering mechanistic understanding of PDAC pathogenesis.

Human intestinal bacteria significantly affect the development of PDAC [147]. Thomas et al*.* demonstrated that antibiotic-induced microbial ablation significantly reduces the incidence of poorly differentiated tumors by 42% in *Kras*-driven PDAC murine models compared with their untreated littermates [19]. In contrast, Chen and colleagues used probiotics (*Lactobacillus paracasei* and *Lactobacillus reuteri*) as adjuvant therapies to effectively suppress PDAC progression in mice [148]. These findings further support the complex influence of the gut microbiota on pancreatic pathology, suggesting its potential for long-range regulation of pancreatic function.

Traditionally regarded as a sterile organ, the pancreas has recently been discovered to harbor symbiotic bacteria that share characteristics with the gut microbiota [23], indicating possible migration through the pancreatobiliary duct. Clinical studies have confirmed significant alterations in the composition of pancreatic and gut microbes in PDAC patients, characterized by the enrichment of specific bacterial taxa [149]. Experimental depletion of gut microbiota in PDAC mouse models has been shown to reduce MDSC infiltration and induce a phenotypic shift in TAMs toward an antitumoral M1-like profile. This process is accompanied by the activation of cytotoxic CD8^+^ T cells and Th1 polarization of CD4^+^ T cells, thereby enhancing antitumor immune responses. These findings indicate that the immunosuppressive effects observed in PDAC models are mediated by the gut microbiota-related TLR2 and TLR5 signaling pathways [150].

Furthermore, TLR9, which is widely expressed on pancreatic epithelial and surrounding stromal cells, exerts multifaceted protumorigenic effects within the pancreatic TME. TLR9 activation stimulates the MAPK, NF-κB, and STAT3 signaling pathways in vivo, leading to the upregulation of key regulatory proteins, including the tumor suppressor p53 and the cyclin-dependent kinase inhibitor p27. Additionally, it enhances the expression of antiapoptotic proteins such as Bcl-2 and Bcl-XL, as well as oncogenic factors like c-Myc and cyclin D1 [151]. TLR9 recognizes unmethylated CpG motifs in gut bacterial DNA, triggering a robust inflammatory response that may contribute to tumorigenesis [152]. These observations suggest that gut microbiota, translocated to the pancreas via the gut‒pancreas axis, may also activate TLR9 signaling in pancreatic cells by releasing CpG-DNA, thereby initiating pancreatic carcinogenesis. Consequently, TLR-dependent signaling pathways position gut microbiota as a promising regulatory target in PDAC.

Notably, probiotic interventions in animal models have demonstrated that engineered *Lactobacillus rhamnosus* GG, encapsulated within a gallium-polyphenol network and chitosan nanocoating, can selectively target and infiltrate PDAC tissues via the gut‒pancreatic axis. This strategy effectively eliminates intratumoral microbiota and LPS, blocks TLR signaling, and enhances the efficacy of PDAC immunotherapy by modulating microbiota‒immune interactions [153].

Although previous studies have focused predominantly on the tumor-promoting effects of bacteria in PDAC tissue samples, fungi are now increasingly recognized as another important risk factor for PDAC [154]. Many studies in both animal models and human individuals have indicated that, in the presence of tumors, pancreatic fungi may migrate from the intestinal lumen and actively participate in the establishment of the TME. Microbiome diversity analysis have revealed that distinct α and β diversity patterns in the fungal microbiome of PDAC tissues compared with those in the gut or healthy pancreas, with a notable enrichment of the genus *Malassezia* [155]. *Malassezia*, a genus traditionally associated with the skin and constituting over 90% of the mammalian skin microbiota, has recently been found to colonize the intestinal niche under homeostatic conditions owing to recent advancements in next-generation sequencing technologies [156]. In mouse pancreatic tissue, *Kras*-driven inflammation induces fungal dysbiosis, which, in turn, triggers the release of pathogens that activate mannose-binding lectin (MBL) and the C3 complement cascade [155]. Complement activation stimulates protumor signaling in tumor-associated leukocytes to establish an immunosuppressive microenvironment [157], thereby accelerating pancreatic carcinogenesis. Despite their relatively low abundance in the gut, fungi are increasingly acknowledged for their role in disease initiation and immune modulation, highlighting the need for further investigation into the mechanisms by which fungi contribute to tumor promotion (Table 1).

**Table 1:** Summary of studies examining the association between different substances and different cancer types

Cancers	Factors	Species	In vivo	In vitro	Reference
HCC	Bacteria	Mouse	+	+	[20, 99]
		Human	-	+	[20]
	Metabolite	Mouse	+	+	[104–106]
		Human	-	+	[107]
BC	Bacteria	Mouse	+	-	[21, 115]
			+	+	[112, 116]
	Metabolite	Human	-	+	[113, 123, 125]
		Mouse	+	+	[125]
LC	Bacteria	Human	-	+	[134]
		Mouse	+	+	[139]
	ncRNAs	Human	-	+	[144, 145]
		Mouse	+	+	[143, 144]
PDAC	Bacteria	Mouse	+	-	[19, 148]
			+	+	[91, 150]
	Fugi	Human	-	+	[155]
		Mouse	+	+	[155]

*Abbreviations*: *HCC* Hepatocellular carcinoma, *BC* Breast cancer, *LC* Lung cancer, *PDAC* Pancreatic ductal adenocarcinoma, *ncRNAs* Non-coding RNAs

In addition to the direct influence of the gut microbiome on PDAC, the intestinal epithelium also participates in pancreatic tumorigenesis through the immune-mediated pathways. Chandra et al*.* demonstrated that the deletion of IL-17 receptor A (IL-17RA) in the gut epithelium leads to dysbiosis, which drives the expansion of Th17 and B cells within tumor tissues. These infiltrating immune cells secrete high levels of IL-17, promoting pancreatic tumor progression by upregulating DUOX2 expression in tumor cells [91]. This substantial evidence provides new insights into how the intestinal epithelium contributes the malignant progression of distant organs through bacterium-immune interactions (Fig. 2).

### Analysis of the intratumoral microbiota

Advances in genomic sequencing have increasingly demonstrated the presence of translocated gut microbes within tumors, characterized by distinct compositions and organ-specific preferences [158]. Gut microbes may leverage host immune mechanisms and disseminate through the bloodstream and lymphatic vessels, particularly in the setting of acute or chronic intestinal barrier disruption. Recent studies suggest that microbiota may be present in precancerous lesions and early stages of tumorigenesis, actively participating in this process through their virulence factors or interactions with other microenvironment components [159, 160].

Intracellular bacteria can reorganize the cytoskeleton of circulating tumor cells, thus enhancing their resistance to fluid shear stress [161]. These microbiota can reshape the TME by modulating the genomic stability of parenchymal cells in tumor tissue, activating oncogenic signaling pathways, inducing chronic inflammation, and suppressing antitumor immune responses [162]. Additionally, the intestinal microbiota contributes to the dissemination and metastasis of cancer cells [159]. The application of microbiota-targeted therapies could offer novel avenues for cancer diagnosis and treatment.

However, the precise origins of intratumoral microbiota have rarely been pinpointed due to several acknowledged limitations, including a low microbial load, the presence of organ-symbiotic microbiota, environmental changes during sampling and detection, and the risk of contamination [163]. Some studies have been challenged the validity of previous conclusions [164–166]. Consequently, there is a pressing need for standardized evaluation protocols to elucidate the exact mechanisms by which translocated gut microbes regulate extraintestinal diseases.

#### Traditional methods

##### 16S rRNA sequencing

16S rRNA gene sequencing has long been established as the gold standard for analyzing the taxonomic composition of bacterial communities. In clinical microbiology, this method is widely utilized for bacterial identification due to its speed and accuracy, achieved by comparing sample sequences with established bacterial databases. Recently, 16S rRNA sequencing has gained significant attention in the field of intratumoral microbiome research. For example, Riquelme and colleagues demonstrated that the α diversity of pancreatic intratumoral bacteria in long-term survivors (≥ 5 years) is significantly higher than that in short-term counterparts [167], suggesting that changes in the diversity of intratumoral bacteria may serve as a predictive marker for survival outcomes.

Currently, most 16S rRNA sequencing methods are based on second-generation sequencing platforms. However, limitations such as primer selection and restricted read length (up to 2 × 300 bp) can hinder diversity assessment and taxonomic resolution, often confining reliable classification to the genus level [168]. Recent advancements in third-generation sequencing technologies, such as full-length 16S rRNA sequencing using PacBio platforms, have enabled the sequencing of entire genes with higher taxonomic resolution. Comparative studies have shown that PacBio sequencing offers superior species-level classification, making it a valuable tool for future high-resolution microbiome research [169]. This technological progress holds promise for accurately detecting low-abundance tumor-associated microbiota, differentiating gut‒origin microbes from environmental contaminants, and elucidating the spatiotemporal relationships between pathogens and tumorigenesis.

##### Polymerase chain reaction

Polymerase chain reaction (PCR) is an in vitro technique that mimics natural DNA replication, allowing for the rapid and targeted amplification of specific genes or DNA sequences. In the field of tumor microbiota research, quantitative PCR (qPCR) and droplet digital PCR (ddPCR) have emerged as indispensable tools for analyzing microbial communities within tumor environments.

qPCR, a highly sensitive and precise molecular biology method, enables the quantitative analysis of DNA or RNA by monitoring fluorescence signals in real time during PCR amplification [170]. In tumor microbiota studies, this technique has been used to quantify microbial nucleic acids in tumor tissues or related samples, providing valuable insights into the functional roles of the microbiota within the TME [171].

ddPCR represents a significant advancement over traditional PCR, offering absolute quantification of microbial DNA in tumor tissues or bodily fluids. This technology achieves unparalleled sensitivity by partitioning the PCR reaction mixture into thousands of nanoliter-sized droplets, each serving as an individual reaction vessel. By analyzing the amplification within each droplet, ddPCR provides a highly efficient and precise method for pathogen detection [172]. Human blood contains trace amounts of cell-free microbial DNA (cfmDNA), which originates from the commensal microbiota. Zozaya-Valdés et al*.* combined 16S rRNA gene sequencing with ddPCR to explore differences in cfmDNA between healthy individuals and patients with metastatic melanoma. Their findings revealed that, after excluding potential contamination, cfmDNA holds promise as a novel biomarker for cancer and other diseases [173].

The application of ddPCR technology in the early stages of tumor development could enable the analysis of specific microbial DNA localization in body fluids and tumor tissues. This approach may help delineate the migration pathways of gut microbiota, offering new perspectives on the dynamic interactions between microbial communities and tumor progression. Such insights could pave the way for innovative diagnostic and therapeutic strategies in oncology.

Although qPCR and ddPCR are powerful tools for tumor microbiota research, they are not without limitations, including susceptibility to PCR inhibitors, complex operational procedures, and the risk of contamination, particularly when handling low-molecular-weight samples, which can significantly compromise detection accuracy [171]. To mitigate these challenges, it is essential to implement stringent contamination control protocols throughout the experimental workflow. This includes the use of dedicated laboratory spaces, rigorous sample handling practices, and appropriate negative controls to ensure the reliability and reproducibility of research findings. By adhering to these measures, the impact of contamination and other technical limitations can be minimized, thereby enhancing the robustness of tumor microbiota studies.

##### Invasion–adhesion-directed expression sequencing

Invasion–adhesion-directed expression sequencing (INVADEseq) is an innovative single-cell RNA sequencing technique that builds upon the standard 10 × Genomics 5′ single-cell RNA sequencing protocol. It incorporates a primer targeting the conserved region of the bacterial 16S rRNA gene alongside a standard primer for eukaryotic poly(A) RNA selection. This dual-primer method enables the generation of DNA libraries that capture both eukaryotic and bacterial transcripts at single-cell resolution. By leveraging 10 × barcodes, INVADEseq can identify individual cells harboring intracellular bacteria, providing a powerful tool for studying host–bacteria interactions at an unprecedented level of detail [174].

The critical advantage of INVADEseq lies in its ability to overcome the limitations of conventional single-cell RNA sequencing methods, which often fail to detect microbial components within the TME. This technique allows for quantification of human cells containing bacteria, the identification of both host cells and intracellular bacterial species, and the elucidation of host transcriptional programs influenced by these bacteria [174]. A major unresolved question in tumor microbiome research is whether tumor-associated bacteria exist freely within tumor tissues or survive intracellularly. INVADEseq addresses this by providing high-resolution identification of bacterial species in the TME and their precise spatial localization, thereby shedding light on bacterial sites of action and their mechanisms of influence. Additionally, this method facilitates cell type-specific classification and comparative analysis of bacteria, enabling researchers to better distinguish the differences and similarities between the tumor microbiome, normal tissue microbiome, and gut microbiota.

Recent studies using INVADEseq have revealed that certain cell types such as macrophages harvested from mucosal tumor samples exhibit a high prevalence of cell-associated bacteria [175]. Given the phagocytic and migratory properties of macrophages, the exact timing of bacterial invasion into these cells remains unclear. However, these findings provide valuable insights into potential initiation sites of bacterial involvement in tumorigenesis. They also raise intriguing questions about whether bacteria initially invade macrophages in extratumoral organs such as the gut and subsequently exert effects on tumor tissues via macrophage migration. Such discoveries highlight the potential of INVADEseq to uncover novel mechanisms of bacterial–host interactions and their implications for cancer biology.

##### Bacteria-based living probes

With the widespread application of bioimaging in visualizing and conducting real-time diagnostics of physiological and pathological processes, bioimaging probes have been extensively developed and utilized. The unique properties of bacteria, including biocompatibility, motility, genetic editability, and ability to target specific sites, make them ideal candidates as engineered living probes for monitoring bacterial activities across different sites [176].

Imageable probes have been successfully used to detect solid tumors via in vivo bioimaging techniques. Noninvasive real-time imaging methods such as MRI and PET use radioactive isotopes or fluorescent dyes to label and trace intratumoral bacteria, enabling researchers to observe their spatial distribution [177, 178]. Additionally, bacteria-based live probe imaging technology has been applied to track the distribution and colonization of gut microbiota in vivo, providing critical insights into their behaviors and functions. Zhang et al*.* developed a novel approach by encapsulating SYTO 9-labeled bioprobes within a colloidal shell composed of amino-modified poly-β-cyclodextrin and tannic acid. This method allowed for real-time monitoring of the mucosal adhesion and colonization rate of encapsulated bacteria in the gut, demonstrating the potential of bacteria-based probes for studying microbial interactions in living systems [179].

Bacteria-based live probes hold immense promise for elucidating the physiological functions and in vivo behaviors of organisms. If a probe could be engineered based on a specific tumor-associated bacterium of gut origin, it could enable tracking and visualization of its localization and distribution in vivo following oral administration. Such a tool would provide insights into its dynamic behavior of these bacteria, potentially uncovering their origins, migration pathways, and functional roles of tumor-associated microbiota. This approach could significantly advance our understanding of the complex interplay between gut microbiota and tumor-associated microbiota, offering new avenues for diagnostic and therapeutic innovation in cancer research.

##### Culturomics

Despite advancements in sequencing technologies for detecting intratumoral microbiota, microbial culture remains the gold standard, as it provides definitive evidence of microbial presence within the TME. Culturomics, a high-throughput culture-based approach, involves cultivating bacterial colonies on diverse culture media under both aerobic and anaerobic conditions to accommodate highly diverse bacterial populations present in tumors. This method not only facilitates the isolation of a large number of culturable microbiota but also enables the discovery of potential novel bacterial species, thereby enriching existing culturable microbial resource libraries.

The key advantage of culturomics is its ability to generate pure microbial cultures, which are essential for further studies on strain characteristics, in vitro modeling, and host‒pathogen interactions [180]. Additionally, culturomics enables the rapid screening of numerous colonies, which can then be identified using MALDI-TOF mass spectrometry or 16S rRNA sequencing [181]. This method provides a direct and reproducible approach for studying tumor microbiota, analyzing microbial diversity and abundance in tumor tissues. Furthermore, it supports subsequent functional studies such as drug sensitivity testing and investigations into pathogenic mechanisms.

Importantly, culturomics offers a precise method for detecting bacterial species, providing definitive evidence regarding the presence of gut microbiota within the tumor microbiome. This makes it a robust tool for diagnosing gut microbial-related diseases and elucidating the role of gut-derived bacteria in tumorigenesis. By combining the strengths of traditional culture methods with modern analytical techniques, culturomics bridges the gap between microbial detection and functional characterization, offering a comprehensive platform for advancing our understanding of the tumor microbiome and its implications for cancer biology and therapy.

#### Emerging technologies and directions

##### 3D tumor model

3D bioprinting is an innovative biofabrication platform that utilizes computer-aided design to precisely deposit living cells, signaling molecules, and biomaterials, creating tissue-engineered structures with highly controlled architectures. These structures can effectively mimic the TME, including patient-derived tumor cell clusters and organoids. By providing microbiota with a survival environment that closely resembles in vivo conditions, 3D bioprinting enables the study of microbial behaviors and their interactions with other elements of the TME [182, 183].

This approach holds significant promise for investigating the involvement of intestinal microbiota in the pathological continuum from chronic inflammation to cancer metastasis in vitro. Specifically, 3D tumor models allow for more in-depth and precise investigations through the coculture of diverse cell types, constructing complex systems that mimic interactions between the gut and extraintestinal tumor tissues.

However, current 3D models may not fully replicate the intricate characteristics of the TME. Further optimization is needed to integrate diverse microbial communities, immune cells, and other critical components of the TME. Moreover, pairing 3D models with in vivo animal studies or clinical patient sample analyses is essential for validating the clinical relevance of research findings. This integrated approach will ensure that insights gained from 3D bioprinting models translate into meaningful advancements in understanding the role of microbiota in cancer biology and developing targeted therapeutic strategies.

##### Artificial intelligence applications

Leveraging the power of artificial intelligence (AI) and machine learning, researchers have made significant strides in precisely categorizing and analyzing microbial DNA and RNA features within specific tumor types, providing new insights for clinical tumor diagnosis and prevention. A landmark study by Poore et al*.* exemplifies this progress, where supervised normalization and decontamination analyses were applied to whole-genome and transcriptome sequencing data from an extensive cohort of 18,116 samples across 33 tumor types and 10,481 patients in the TCGA database. By meticulously mining microbial reads from these samples, the team constructed a comprehensive tumor microbiome dataset. AI-driven approaches were then utilized to identify and differentiate microbial features among various tumor types and to evaluate their predictive performance. Although subsequent evaluations revealed that the study initially overestimated microbial reads, casting some doubt on the validity of its findings [166], refined microbial analysis and screening methodologies corroborated similar conclusions [184]. This suggests that AI can amplify small errors, which can be fatal to the accuracy of the results. The establishment of rigorous inclusion and exclusion criteria can mitigate these risks, ensuring more reliable outcomes from machine learning applications. Thus, AI may offer a promising avenue to advance cancer-associated microbiome studies toward a more precise era.

**Fig. 3:**
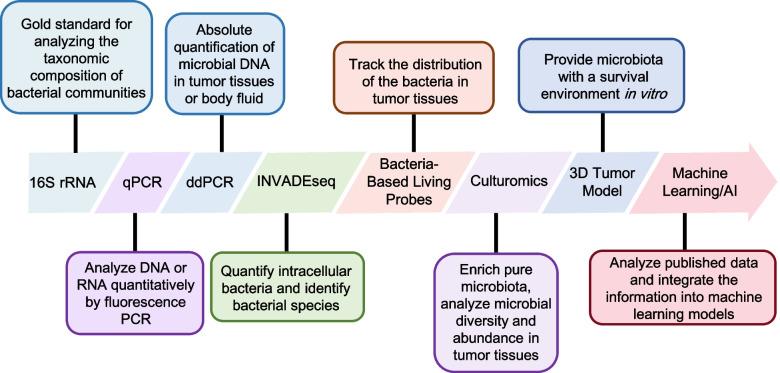
Overview of methodological advances in intratumoral microbiota analysis. Emerging technological approaches are being developed to elucidate the spatiotemporal dynamics and functional relationships between intestinal dysbiosis, intratumoral microbiota, and cancer progression, encompassing both spatial localization and temporal evolution of microbial communities within tumor microenvironments

A major challenge in analyzing intratumoral microbiota is sample contamination, which can obscure true microbial signals. AI offers a transformative solution by facilitating the development of sophisticated decontamination algorithms that enhance data reliability, sensitivity, and specificity. While computer simulations cannot fully replicate the behaviors of the microbiota in real-world settings, AI can analyze published datasets and integrate this information into machine learning models. By comparing variations in gut microbiota with changes in tumor microbial abundance and pathological alterations, AI can uncover potential spatiotemporal relationships. This approach may reveal that the tumor-associated microbiota originates from the gut, providing insights into the timing and mechanisms of microbial involvement in tumorigenesis. Furthermore, the integration of 3D models with AI-based data analysis techniques could offer precise interpretation of microbiota behaviors at different stages of tumors, guiding the design of targeted experiments to test these hypotheses (Fig. 3).

Clinical investigations into the intratumoral microbiota represent an important initial step, yet there remains a pronounced lack of comprehensive studies elucidating their roles in tumorigenesis and therapeutic responses. The field is marked by significant knowledge gaps, necessitating intensive exploration and rigorous scientific validation. Given the intricate relationship between gut microbiota, the emergence of intratumoral microbiota, and their dynamic interplay with the TME, targeting gut microbiota and developing relevant clinical intervention strategies could significantly advance this emerging field. Such efforts may pave the way for novel diagnostic and therapeutic approaches, ultimately improving patient outcomes in oncology.

### Perspectives and future challenges

The causal relationships between gut dysbiosis and extraintestinal cancers are emerging as a focal point of research, shedding light on the profound influence of the gut microbiome on cancer development beyond the intestines. Both clinical trials and in vivo animal experiments have convincingly demonstrated that specific gut microbiota can modify disease phenotypes by compromising the integrity of the intestinal barrier, modulating immune responses, and influencing metabolic pathways. These findings underscore the gut microbiome's pivotal role in shaping systemic health and disease.

Pinpointing a translocated microbiome is a commonly stated priority and expectation for contemporary detection methods. However, relying solely on a straightforward analysis of the relative abundance of taxa may overshadow on other critical aspects, such as the origin and functional activity of intratumoral microbiota. To address this limitation, metabolomics techniques are now emerging as a highly appealing option for enhancing our capacity to decipher the intricate host-microbiome interactions. This indicates that pairing metagenomic data with metabolomics experiments could be more effective than the relative abundance alone, opening new avenues for exploring the functional dynamics of the microbiome in cancer biology. This integrative approach holds great promise for advancing the field and uncovering novel therapeutic targets.

Beyond its role in systemic cancers, gut microbiota is increasingly recognized as a key player in the gut-brain axis, influencing brain homeostasis and offering new perspectives for understanding and managing brain tumors. This bidirectional communication between the gut and the brain highlights the microbiome's far-reaching impact on health and disease. Additionally, the therapeutic potential of probiotics in combating various cancers has garnered significant attention. Recent preclinical and clinical studies have highlighted the supportive role of probiotic-based treatments in antitumor strategies, raising optimism for their integration into cancer therapy.

In summary, the gut microbiota engages in complex and multifaceted interactions with extraintestinal tumors. Deciphering the molecular communication that regulates these interactions holds the potential to uncover the underlying mechanisms of tumorigenesis and progression. This, in turn, can establish a solid foundation for the development of tailored anticancer therapeutics.

## Data Availability

Not applicable.

## Abbreviations

3-IPA: 3-Indolylpropionic acid
AI: Artificial intelligence
Akt: Protein kinase B
ALD: Alcoholic liver disease
BC: Breast cancer
cfmDNA: Cell-free microbial DNA
circRNA: Circular RNA
CLD: Chronic liver disease
CRC: Colorectal cancer
*Crb1*: *Crumbs homolog 1*
DCA: Deoxycholic acid
DCs: Dendritic cells
ddPCR: Droplet digital PCR
DSS: Dextran sulfate sodium
EBV: Epstein–Barr virus
Fut2: Fucosyltransferase 2
GLA: Gut‒lung axis
GVB: Gut vascular barrier
*H.h.*: *Helicobacter hepaticus*
HCC: Hepatocellular carcinoma
HCV: Hepatitis C virus
HMGB1: High-mobility group Box 1
HSCs: Hepatic stellate cells
HPV: Human papilloma virus
IBD: Inflammatory bowel disease
IBS: Irritable bowel syndrome
ICB: Immune checkpoint blockade
IFN-γ: Interferon-γ
IL: Interleukin
IL-17RA: IL-17 receptor A
INVADEseq: Invasion–adhesion-directed expression sequencing
JAMs: Junctional adhesion molecules
LC: Lung cancer
LPA: Lysophosphatidic acid
LPC: Lysophosphatidylcholine
LPS: Lipopolysaccharides
LTA: Lipoteichoic acid
LUAD: Lung adenocarcinoma
MBL: Mannose-binding lectin
MDSC: Myeloid-derived suppressor cell
miRNA: MicroRNA
mMDSCs: Monocytic myeloid-derived suppressor cells
MAFLD: Metabolic-associated fatty liver disease
MLCK: Myosin light chain kinase
NASH: Nonalcoholic steatohepatitis
ncRNAs: Non-coding RNAs
NSCLC: Non-small cell lung cancer
PAMPs: Pathogen-associated molecular patterns
PCR: Polymerase chain reaction
PDAC: Pancreatic ductal adenocarcinoma
PGE: Prostaglandin E
PI3K: Phosphoinositide 3-kinase
PRRs: Pattern recognition receptors
PV-1: Plasmalemma vesicle-associated protein-1
qPCR: Quantitative PCR
SASP: Senescence-associated secretory phenotype
SCFAs: Short-chain fatty acids
SCLC: Small-cell lung cancer
T3SS: Type III secretion system
TCA: Taurocholic acid
Th17: Helper T 17
TGF: Transforming growth factor
TJs: Tight junctions
TLR: Toll-like receptor
TME: Tumor microenvironment
TNF-α: Tumor necrosis factor-α
Tregs: Regulatory T cells
VDR: Vitamin D receptor
ZO-1: Zonula occludens-1
